# Assessing the Claims of Participatory Measurement, Reporting and Verification (PMRV) in Achieving REDD+ Outcomes: A Systematic Review

**DOI:** 10.1371/journal.pone.0157826

**Published:** 2016-11-03

**Authors:** Sandra Hawthorne, Manuel Boissière, Mary Elizabeth Felker, Stibniati Atmadja

**Affiliations:** 1 Centre for International Forestry Research, Bogor, Indonesia; 2 Centre de coopération Internationale en Recherche Agronomique pour le Développement, Montpelier, France; Pacific Northwest National Laboratory, UNITED STATES

## Abstract

Participation of local communities in the Measurement, Reporting and Verification (MRV) of forest changes has been promoted as a strategy that lowers the cost of MRV and increases their engagement with REDD+. This systematic review of literature assessed the claims of participatory MRV (PMRV) in achieving REDD+ outcomes. We identified 29 PMRV publications that consisted of 20 peer-reviewed and 9 non peer-reviewed publications, with 14 publications being empirically based studies. The evidence supporting PMRV claims was categorized into empirical finding, citation or assumption. Our analysis of the empirical studies showed that PMRV projects were conducted in 17 countries in three tropical continents and across various forest and land tenure types. Most of these projects tested the feasibility of participatory measurement or monitoring, which limited the participation of local communities to data gathering. PMRV claims of providing accurate local biomass measurements and lowering MRV cost were well-supported with empirical evidence. Claims that PMRV supports REDD+ social outcomes that affect local communities directly, such as increased environmental awareness and equity in benefit sharing, were supported with less empirical evidence than REDD+ technical outcomes. This may be due to the difficulties in measuring social outcomes and the slow progress in the development and implementation of REDD+ components outside of experimental research contexts. Although lessons from other monitoring contexts have been used to support PMRV claims, they are only applicable when the enabling conditions can be replicated in REDD+ contexts. There is a need for more empirical evidence to support PMRV claims on achieving REDD+ social outcomes, which may be addressed with more opportunities and rigorous methods for assessing REDD+ social outcomes. Integrating future PMRV studies into local REDD+ implementations may help create those opportunities, while increasing the participation of local communities as local REDD+ stakeholders. Further development and testing of participatory reporting framework are required to integrate PMRV data with the national database. Publication of empirical PMRV studies is encouraged to guide when, where and how PMRV should be implemented.

## Introduction

Reducing emissions from deforestation and forest degradation and enhancing carbon stocks (REDD+) is a mechanism to reduce carbon emissions from tropical forests in developing countries [1]. As REDD+ is based on performance-based incentives [2], a measurement, reporting and verification (MRV) system of carbon stocks is required to assess the effectiveness of REDD+ outcomes. However, accurate estimation of changes in carbon stocks requires a ground-based carbon inventory that can be costly to obtain [3]. In addition to carbon reduction outcomes, there is an increasing expectation that social and environmental effects of REDD+ are monitored to ensure safeguards are implemented and co-benefits are maximized [4]. This will require the collection of non-carbon data, such as biodiversity, livelihood benefits and drivers of deforestation and degradation, that can potentially be incorporated into the MRV system [5–7].

Participatory MRV (PMRV) is the involvement of local communities in MRV activities within the REDD+ context. Participatory or community-based measurement of biomass to estimate carbon stocks is the most common implementation of PMRV to date [8–16]. We define participatory reporting as the involvement of local communities in reporting carbon stock data for its integration into a national monitoring system, such as a national forest inventory. Participatory verification is defined here as the involvement of local communities in verifying carbon stocks or land use change, such as the use of participatory forest mapping to improve the stratification of vegetation types in remote sensing data or local monitoring of land use to signal ground changes in near real-time [15, 17, 18]. Several studies suggest that local communities can also collect non-carbon data required for monitoring REDD+ safeguards and co-benefits [5, 6, 9, 15, 19, 20].

PMRV has been promoted as a strategy to achieve various outcomes that support REDD+ implementation, such as lowering the transaction cost of MRV and increasing the participation of local communities and indigenous people [6, 9, 10, 20, 21]. In response to these claimed benefits, there have been increased number of PMRV implementations in REDD pilot projects and interest in integrating PMRV into a national MRV system [13, 18–20, 22, 23]. There is, however, a need to examine the evidence of PMRV's role in achieving REDD+ outcomes as some of the claims have been derived from other participatory initiatives, such as community-based forest management or biodiversity monitoring [5, 6, 10]. A review of current knowledge on PMRV will also be pertinent due to the increasing number of studies on participatory carbon monitoring in recent years [8, 11, 15, 24–27].

This systematic review examines the claims of PMRV's role in achieving REDD+ outcomes that are effective in carbon emission reduction, cost efficient, equitable to all countries and generating multiple benefits. It builds on our previous study that identified strategies and conditions that support PMRV implementation in the literature [28]. This review is presented in several sections. In the method section, we describe the process used to identify and analyze PMRV publications. The results section outlines the characteristics of PMRV publications, PMRV claims of achieving REDD+ outcomes identified from the literature, analysis of evidence supporting PMRV claims and the context of citations used as supporting evidence. We discuss recent implementation of PMRV and current knowledge gaps, empirical evidence supporting PMRV claims and study limitation in the discussion section. In conclusion, we recommend ways to address the knowledge gaps.

## Materials and Methods

We applied the search terms “REDD+ AND (participatory OR local OR community) AND monitor” for all publications published up to September 2014. A starting year was not used in the search to capture all the PMRV-related publication. The search was conducted using the following databases and publication collections:

- ISI Web of Science.
- REDD desk (http://theredddesk.org).
- REDD+ community (http://reddcommunity.org).
- Community Carbon Forestry (http://www.communitycarbonforestry.org).
- Centre for International Forest Research (http://www.cifor.org).

The listed databases and collections were selected because they function as the repositories of knowledge on REDD+ and community-based forest management.

We have included non peer-reviewed publications (*grey literature*) in our literature search. This type of literature can play an important role in documenting the accumulation of knowledge and supporting the development and dissemination of recent works, providing a valuable source of up-to-date, field-based PMRV experience. Meta-analyses that exclude non peer-reviewed publications are likely to over-represent larger studies with statistically significant results, resulting in the inflation of effect size [29]. For the purpose of this study, we applied a similar analysis to peer-reviewed (PRP) and non peer-reviewed publications (NPRP), while acknowledging the potential lack of rigor of the latter as they have not gone through the peer-review process. Presentation of our results has been designed to distinguish between the two knowledge sets.

Based on the title and/or abstract of the search results, we identified publications in English that primarily describe, analyze and discuss participatory MRV activities in the context of REDD+. We excluded the following items from the results:

- Newsletters, news articles, theses, technical manuals and non-electronic book chapters.
- Publications focusing on the development of MRV method or system.
- Publications focusing on community participation outside of the MRV context, such as participation in REDD+ implementation or community-based forest management.
- Publications describing project results that have been reported elsewhere.

**Fig 1:**
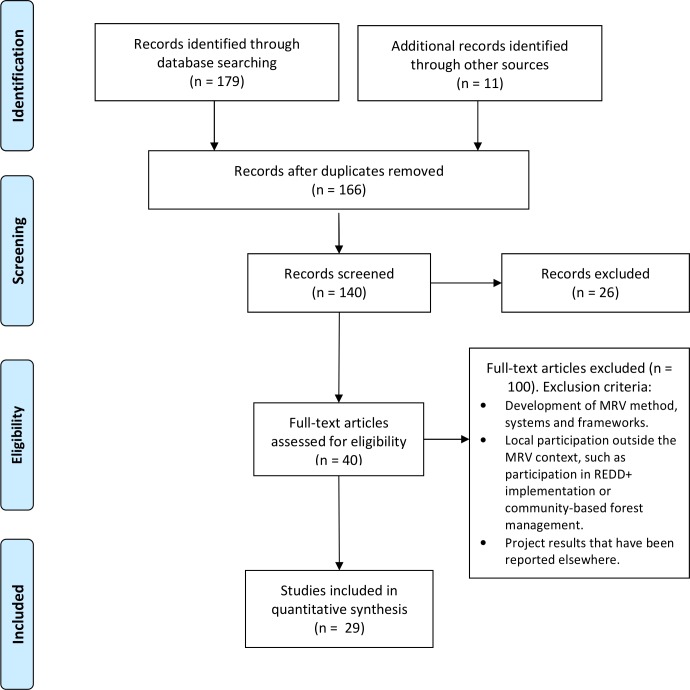
Selection process of PMRV publications described in a Preferred Reporting Items for Systematic Reviews and Meta Analyses (PRISMA) flow diagram.

We also excluded our previous literature review from this analysis [28]. The references of the relevant PMRV publications were used to identify additional publications. We included nine publications from a special issue of *Forests* on community monitoring in MRV [30], despite four of them being published after September 2014. The selection process is described in a Preferred Reporting Items for Systematic Reviews and Meta Analyses (PRISMA) flow diagram (Fig 1), while the PRISMA checklist can be found in S1 Table.

Each PMRV publication is characterized based on the following attributes: publication type (PRP, NPRP), study type (empirical, non-empirical) and publication year. Study type is used to examine the relative contributions of field based experiences and desk-studies to the development of PMRV discourse. The categories of study type are defined as follows: an empirical study reports and analyzes data from field implementation of PMRV activities, a non-empirical study synthesizes or reviews the literature on the design and implementation of PMRV.

For each PMRV publication, we used MaxQDA software (MaxQDA version 11, Verbi GmbH, Germany) to encode statements that describe the potential or demonstrated effects of PMRV on REDD+. These statements were listed as REDD+ outcomes. For example, a statement that links local monitoring of carbon stocks with securing access to forest resources is considered as a PMRV claim that achieves a REDD+ outcome of securing access to forest resources. We examined the supporting evidence for each PMRV claim using the following categories:

- Empirical finding supporting evidence (EFSE): original finding or analysis supporting the claim is presented in the publication. This is considered as a strong evidence for the claim.
- Citation supporting evidence (CSE): citation(s) to other publication(s) is used to support the claim. This indicates some evidence for the claim may have been established.
- Assumption (AS): a citation nor finding to support the claim has not been provided in the publication. The claim is considered as a potential role of PMRV.

Each PMRV claim in a publication can be supported with EFSE and/or CSE; alternatively, AS is assigned to a PMRV claim in the absence of EFSE or CSE in the text.

To help us understand how PMRV claims draw upon lessons from other contexts, we examined CSE from peer-reviewed publication (PRP) further. Each CSE may correspond to one or multiple publications (references). The context of each cited publication was determined based on the main topic identified from its title or abstract. These contexts have been broadly grouped into PMRV, participatory monitoring, REDD+ implementation, forest management and participatory GIS.

## Results

### Characteristics of PMRV publications

We identified 29 PMRV publications that were published between 2009 and 2014, which include 20 PRP and 9 NPRP (Table 1). Empirical studies made up nearly half of the identified PMRV publications (14 out of 29).

**Table 1:** Classification of PMRV publications based on publication and study types.

Publication type		Study type			Total
	Empirical study	References	Non-empirical study	References	
Peer-reviewed	11	8–10, 13, 14, 16, 22, 24, 26, 27, 31	9	5, 6, 18–21, 23, 32, 33	**20**
Non peer-reviewed	3	12, 15, 25	6	11, 34–38	**9**
**Total**	**14**		**15**		**29**

**Fig 2:**
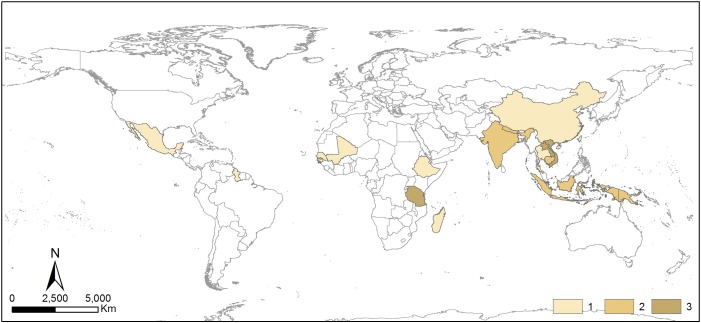
Number and locations of PMRV projects (17 countries, 28 projects) from PMRV empirical studies analyzed in this review.

Based on PMRV empirical studies, we identified 28 PMRV projects in 17 countries across three tropical continents (Fig 2). There was a concentration of projects in southeast Asia (Laos and Vietnam) and Tanzania. Several publications [11, 16, 22] refer to the Kyoto: Think Global Act Local (K:TGAL) project that was conducted in seven countries: Mali, Senegal, Guinea Bissau, Tanzania, Nepal, India and Papua New Guinea. Torres et al. [27] reviewed eleven sub-national REDD+ projects in Mexico, but they concluded that only one project had the capacity to readily implement PMRV activities. Based on this, we listed one PMRV project in Mexico.

A summary of the characteristics of PMRV projects is presented in Table 2. PMRV projects spanned a wide range of forest types from tropical forests to savannah woodland. Land tenure system varied from community-managed to state-managed. Nineteen projects engaged the local communities in carbon stock measurements, while ten projects collect additional information such as forest (land use) change, use of forest resources, biodiversity and social data. The participation level of local communities in most PMRV projects has been limited to data gathering (i.e. Category 2–3 in monitoring schemes, see [39]) as those projects were conducted to test the feasibility of PMRV. Several projects incorporated PMRV into the local implementation of REDD+ projects or land-use climate mitigation actions [25, 27, 31], which have enabled greater level of community participation.

**Table 2:** Summary characteristics of PMRV empirical studies identified in the literature search.

Publication	Location	Forest type and tenure*	Sample size	Data collected	Analytical methods*
**Peer-reviewed**					
Bellfield et al. [24]	Guyana	Old growth tropical forest, savannah, wetland	16 communities, 117 plots in forests, 128 plots in agricultural areas	• Drivers of deforestation and forest degradationBiomass inventory • Mapping of agriculture • Ground truth data of vegetation type and land use • Resource use • Household social and economic data	Comparison of mean biomass obtained by local community and published estimates. Comparison of disturbed areas obtained by local community and remote sensing data.
Brofeldt et al. [8]**	• Indonesia • China • Laos • Vietnam	• Indonesia: lowland dipterocarp (community forests) • China: tropical mountain (collective and State forests) • Laos and Vietnam: evergreen monsoon (State forests with local user rights)	9 villages, 135 plots	• Biomass inventory • Cost of monitoring (e.g. transport, salaries, training material, fieldwork, etc.)	Comparison of mean biomass obtained by local community and professional foresters using t-test and Wilcoxon’s signed rank test.
Danielsen et al. [10]	• India • Tanzania Madagascar	• India: oak and pine • Tanzania: miombo woodland and montane evergreen • Madagascar: dry deciduous	19 sites, 125 plots for carbon stocks, 90 forest utilization surveys (each over 3-month period)	• Biomass inventory • Forest utilization (number of cut trees)	Comparison of mean biomass and forest utilization obtained by local community and professional foresters using aired t-test. Power analysis was used to estimate the number of required sampling plots
Danielsen et al. [9]**	• Indonesia • China • Laos • Vietnam	• Indonesia: lowland dipterocarp (community forests) • China: tropical mountain forest (collective and State forests) • Laos and Vietnam: evergreen monsoon forest (State forests with local user rights)	9 villages, 289 plots	• Biomass inventory • Cost of monitoring (e.g. transport, salaries, training material, fieldwork, etc.)	Comparison of mean biomass, variance biomass, tree girth and plot demarcation obtained by local community and professional foresters using paired t-test, F test and Wilcoxon’s signed rank test. Comparison of mean annual cost.
Mukama et al. [13]	Tanzania	Dry, riverine forest and wet miombo (community managed forests)	3 villages, 261 plots,	• Demographic • Importance of forest management • Biomass inventory • Forest mapping and stratification	Comparison of mean biomass obtained by local community and published estimates.
Pratihast et al. [14]	Vietnam	Tropical forest	1 commune, 17 biomass plots and 48 disturbance monitoring plots	• Biomass inventory • Disturbance events (areas, timing, type)	Comparison of mean biomass obtained by local community and professional foresters using simple linear regression and Index of Agreement (IA). Comparison of forest disturbance area and timing estimated by local community against remote sensing data.
Pratihast et al. [26]	Ethiopia	Afro-montane cloud forest	1 reserve, 30 local experts from 10 administrative units	REDD+ activity data recorded with mobile device with integrated GPS: • Forest degradation • Deforestation and reforestation	Comparison of locally collected REDD+ activity data against field-based reference dataset (FRD) and remote sensing (RS). Measures of accuracy: • Error matrix for spatial categories and GPS error • Time lag for temporal data • Error matrix for thematic data
Shrestha et al.[31]	Nepal	High, medium and low altitude forested watersheds (community forests)	112 community forests, 570 plots	• Annual increment of carbon stocks • Demographic data • Drivers of deforestation and degradation	Review of project document and auditing results to report on governance structure, carbon stock enhancement, benefit sharing mechanism and additional activities to ensure additionality and prevent leakage.
Skutsch and Ba [16]	• Mali • Senegal Guinea Bissau	Tropical dry forest and savannah woodlands (community forests)	19 villages, 260 plots	• Annual biomass inventory	Not available
Skutsch et al. [22]	• Tanzania • India • Nepal	• Tanzania: miombo woodland (community forests) • India: oak and pine (community forests) • Nepal: oak (community forests)	2 villages, 1 reserve	• Biomass inventory • Cost of monitoring (salaries, training material, travel, etc.)	Comparison of mean biomass obtained by local community and professional foresters.
Torres et al. [27]	Mexico	Deciduous, evergreen and cloud (community forests, nature reserves)	rojects	• Project proposal • Survey on MRV implementation	Multi-criteria analysis ranking based on project proposal.
**Non peer-reviewed**					
Brewster et al. [25]	Cambodia	Forest type not specified (community forests)	13 community forest, 120 permanent plots	• Social assessment • Biomass inventory • Biodiversity assessment • Forest change	Not available
Khoa [12]	• Laos • Thailand • Vietnam	Forest type not specified (conservation and community forests)	5 communities, 31 villages	• Resource use • Local capacities to participate in MRV • Institutional support	Not available
Schevens [15]	• Papua New Guinea (PNG) • Cambodia • Indonesia • Laos • Vietnam	• PNG: lowland and montane primary moist tropical forest • Cambodia: deciduous forest • Indonesia: dryland woodlots and home gardens	Not available	• Forest monitoring and biomass inventory (PNG, Cambodia, Indonesia). • Biomass inventory of living tress deadwood (Cambodia) • Participatory mapping of vegetation types	Comparison of mean biomass obtained by local community and published estimates.

*when information is available.

**projects conducted at the same sites.

**Fig 3:**
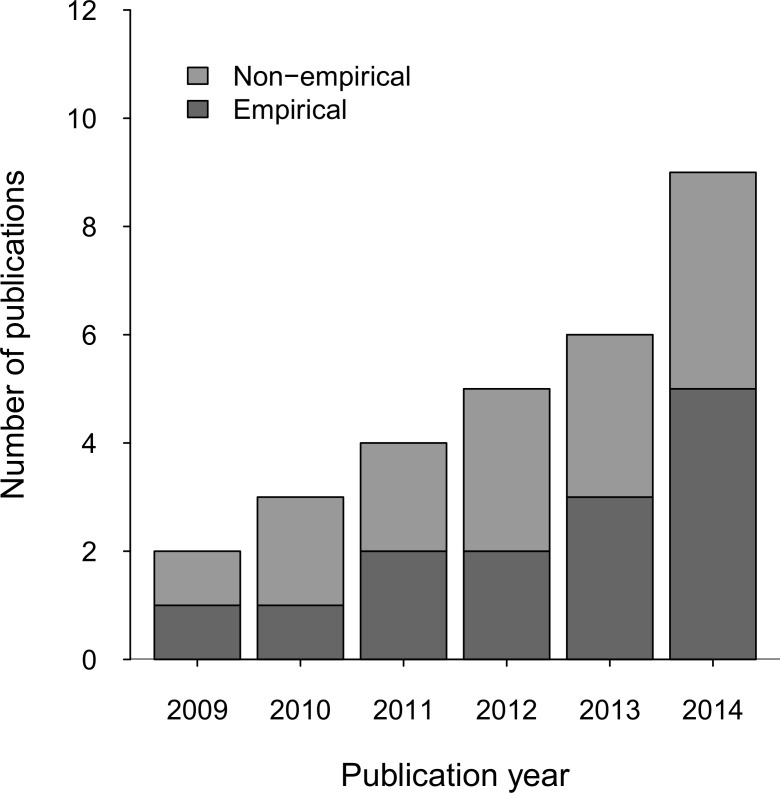
Number of PMRV publications categorized by study type.

The number of PMRV publications has increased steadily from 2009 onwards (Fig 3), which peaked in 2014 due to a special issue in the journal *Forests* on the potential role of community monitoring in MRV. The proportion of empirical to non-empirical studies also increased slightly over time.

### Identifying PMRV claims

The potential and demonstrated effects of PMRV on REDD+ outcomes were summarized in 24 PMRV claims (Table 3). The REDD+ outcomes have been categorized into social and technical outcomes, although a few REDD+ outcomes are related to both. Social REDD+ outcomes refer to outcomes that affect local communities, while technical outcomes refer to those that affect the implementation of MRV system or REDD+. PMRV claims were also labeled based on whether they support, hinder or highlight a requirement to achieve REDD+ outcomes.

**Table 3:** Summary of PMRV claims that support, hinder and highlight a requirement to achieve REDD+ outcomes as identified in PMRV literature.

REDD+ aspect	REDD+ outcome	Claims of PMRV's effect on REDD+ outcome		Reference
		PMRV effect^1^	Description	
Social	Community access to resources	(+)	Access to forest resources are secured and legitimized as part of the PMRV framework.	6, 8–10, 12, 25, 27, 31, 32, 34, 37
Social	Equitable benefit sharing	(+)	Communities' claim to REDD+ financial opportunities is strengthened, while transparency in benefit sharing is improved.	5, 6, 9, 10, 14, 15, 20, 22, 25, 31–34, 38
Social	Equitable benefit sharing	(-)	Funds may not reach the people who undertake the PMRV activities as a result of mismanagement, corruption or elite capture.	6, 8, 19, 32
Social	Environmental awareness	(+)	Local communities have greater environmental awareness, e.g. greater understanding of forest ecosystem functions, that leads to more sustainable forest management.	5, 10, 23, 25, 31
Social	Forest management	(+)	Monitoring data improves decision-making in forest management and enables rapid management response.	5, 6, 9–12, 20–27, 31–34, 36–38
Social	Governance and institutions	(+)	Accountability, transparency and enforcement of regulations in managing local forest resources are increased.	6, 9, 23, 26, 31–34, 37, 38
Social	Governance and institutions	(*)	Supports from local agencies and institutions, sub-national and national government are required.	6, 9, 10, 12, 13, 23–25, 27, 31–33
Social	Local engagement and/or ownership	(+)	Commitment and support from local communities for REDD+ programs are increased, e.g. greater participation or less conflict in REDD+ implementations.	5, 6, 9, 14, 15, 18, 20, 21, 23–27, 31, 33–37
Social	Local engagement and/or ownership	(-)	Involvement of local communities in MRV is often limited to data gathering.	6, 9, 21, 24, 25, 27, 34, 35
Social	Stakeholder relationship	(+)	Relationships are built and cooperation improved between local communities and other stakeholders.	5, 6, 15, 20, 21, 23, 25, 34, 35, 37, 38
Social	Stakeholder relationship	(*)	Conflicts about resource access and poor relationships between stakeholders must be addressed prior to implementing PMRV.	5, 24, 25, 27, 31, 33
Social and Technical	Enhancement of co-benefits	(+)	Availability of data and local engagement through PMRV can enhance REDD+ co-benefits, such as biodiversity conservation and livelihood improvement.	5, 6, 10, 13, 15, 16, 18, 20–23, 25, 27, 31–35, 37, 38
Social and Technical	Safeguards implementation	(+)	REDD+ social and environmental safeguards (e.g. biodiversity protection, full participation of local communities) are implemented.	8–11, 15, 20, 21, 24, 31, 33–38
Technical	Measurement accuracy	(+)	Local data can be as accurate as professional survey.	5, 6, 8–11, 14–16, 18–27, 31–33, 35, 36
Technical	Measurement accuracy	(-)	Variations in the skills and motivation can result in less precise measurements.	5, 6, 8–10, 13, 16, 24–26, 33, 34, 37
Technical	Accurate reporting	(-)	Linking payment to monitoring results can create an incentive to report false or inflated results.	6, 9, 10, 19
Technical	Accurate reporting	(*)	Capacity for reporting and rigorous reporting system must be developed.	5, 21, 24, 25
Technical	Cost effectiveness	(+)	PMRV costs less than professional survey in obtaining local data.	5, 6, 8–11, 13–16, 20, 22–26, 31–38
Technical	Cost effectiveness	(-)	Time and resources devoted to PMRV activities may be greater than direct benefits of PMRV for local communities.	6, 8, 10, 13, 23–27, 31, 32, 34, 35, 37
Technical	Incorporation of local knowledge	(+)	Local knowledge can improve quality of PMRV data, e.g. providing real time data of forest changes, improving tree species identification.	6, 9, 10, 13, 15, 23–26, 33, 34, 36–38
Technical	Measurement frequency	(+)	Proximity between local communities and the forest enables repeated measurements to be conducted.	8, 10, 15, 24, 26, 33–36
Technical	Non-carbon data monitoring	(+)	Local communities can identify local drivers of land use change as well as monitoring social, economic and ecosystem indicators of REDD+ impact.	5, 6, 9, 10, 12, 14, 15, 18, 20, 21, 24–27, 33–38
Technical	Replicability	(-)	Scaling up locally-based PMRV to the national level can be challenging. Application of a uniform standard may be impractical due to variation in local conditions.	5, 24, 25, 31, 36, 37
Technical	Verification of remote sensing data	(+)	Local data (e.g. carbon stocks, land use change) can be used to calibrate or verify remotely sense data.	5, 9, 12, 14, 15, 18, 20, 22, 24–26, 31, 34, 38

^1^ (+) = supporting REDD+ outcome, (-) = hindering REDD+ outcome, (*) = required for achieving REDD+ outcome.

The discussion of PMRV effects on REDD+ has primarily focused on the potential for PMRV to support REDD+ outcomes. However, there were PMRV claims that point out the challenges in achieving REDD+ outcomes. For example, PMRV can be the basis for benefit sharing mechanisms and strengthen local claim to REDD+ benefits, but its implementation can burden those participating in PMRV without providing their fair share of REDD+ benefits, such as when there is poor local governance or unfair benefit sharing mechanisms [6, 32]. Other REDD+ outcomes with contradictory evidence are cost effectiveness [6, 8, 10, 13, 23–26, 31, 32, 34, 35, 37], measurement accuracy [5, 6, 8–10, 15, 16, 24–26, 33] and local engagement and/or ownership [6, 9, 21, 24, 25, 27, 34, 35].

### Analysis of evidence supporting PMRV claims

**Fig 4:**
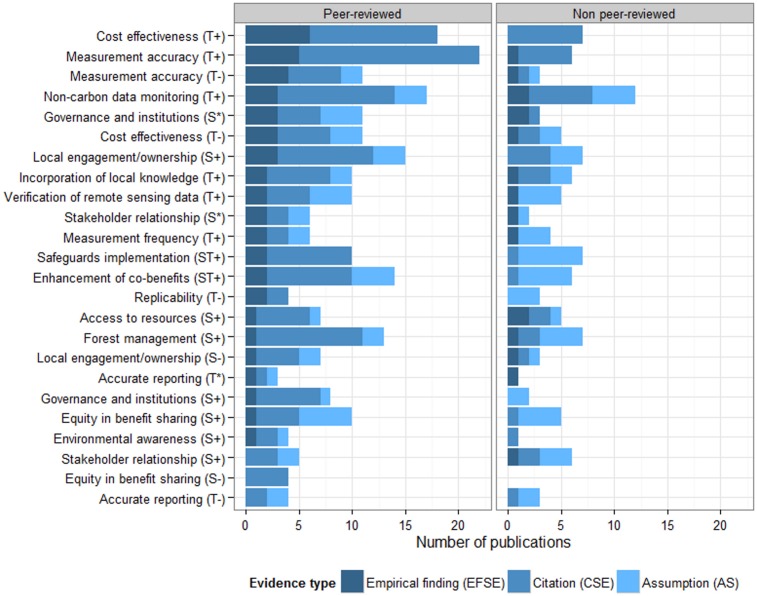
Number of peer-reviewed and non peer-reviewed publications supporting PMRV claims with empirical finding (EFSE), citation (CSE) and assumption (AS). **The claims are listed in descending order based on the number of empirical finding in peer-reviewed and non peer-reviewed publications.** Letters T and S next to PMRV claims represent technical and social aspects of REDD+ outcome respectively. Positive (+), negative (-) or asterisk (*) signs represent claims that support, hinder/challenge and highlight requirement for achieving REDD+ outcomes respectively.

In this section, we examine the number of publications and type of evidence supporting PMRV claims (Fig 4). Most of PMRV claims have been supported with one or more empirical finding (EFSE). Two PMRV claims with the highest number of EFSE (Fig 4) were lowering MRV costs (EFSE: 6 PRP [8–10, 13, 14, 22]) and obtaining accurate local measurement (EFSE: 5 PRP [8–10, 22, 26] and 1 NPRP [15]). Other REDD+ outcomes well supported by empirical finding include obtaining local non-carbon data (e.g. drivers of deforestation and degradation, resource use, biodiversity, socio-economic) and increased local engagement with REDD+ process. Two PMRV claims were without EFSE: the risks of inequality in benefit sharing (CSE: 4 PRP [6, 8, 19, 32]) and inaccurate (false) reporting (CSE: 2 PRP [9, 19] and 1 NPRP [37]; AS: 2 PRP [6, 10] and 2 NPRP [25, 34]). Most of PMRV claims related to REDD+ social outcomes (8 out of 11 claims) were supported by two or fewer EFSE compared to those related to REDD+ technical outcomes (3 out of 11 claims), suggesting they have been assessed less frequently than the technical outcomes.

### Context of citations supporting PMRV claims

**Fig 5:**
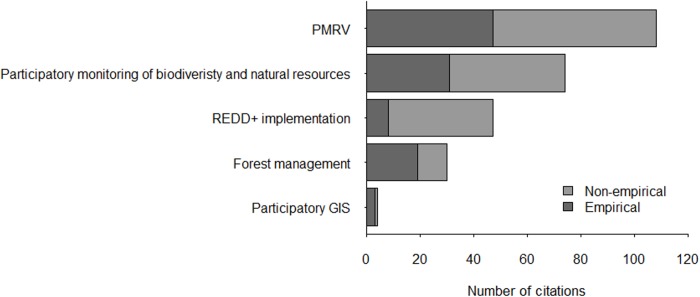
Contexts of citation supporting PMRV claims in the peer-reviewed publications.

Citation makes up most of the supporting evidence for PMRV claims so we have analyzed the context of citation in PRP. We identified 134 occasions when citations were used to support PMRV claims (CSE) in 20 PRP, which yielded a total of 259 citations. The context of each citation has been classified into one of the following: PMRV, participatory monitoring of biodiversity and natural resources, REDD+ implementation, participatory forest management and participatory GIS (Fig 5).

Out of 259 citations used as CSE, 104 citations (40%) refer to 33 publications with PMRV context, including 14 publications that are analyzed in this study. Non-empirical studies make up a larger proportion (58%) of the citation sources than empirical studies. The three most cited publications that we have analyzed here are Danielsen et al. [10], Pratihast et al. [18] and Pratihast et al. [14] with 13, 12 and 11 citations respectively.

More than half of all citations (155 citations) refer to publications with research topics outside of the PMRV context. The non-PMRV context that yield the most citations is participatory monitoring of biodiversity and natural resources (74 citations), followed by REDD+ implementation (47 citations), participatory forest management (30 citations) and participatory GIS (4 citations).

Our analysis also shows that two authors have been influential in PMRV research: Danielsen [8–10, 32] and Skutsch [6, 10, 16, 18, 19, 22, 35, 36]. In addition to publishing works in the PMRV context, they have contributed to the development of participatory (local) monitoring and community forestry discourse respectively. They produced a total of 29 publications that have been used extensively to support PMRV claims (113 citations).

## Discussion

### Recent implementation of PMRV

PMRV projects have been conducted to monitor carbon and non-carbon data in a wide range of geographical areas, forest types and land tenure systems. Yet, there are some notable gaps in current PMRV implementation.

The number of PMRV projects located in Latin America was surprisingly lower than the other continents (Fig 2). For example, we did not identify an empirical PMRV project from Brazil in our literature search despite its early adoption of REDD+ and substantial carbon market [40] as well as the commitment of its government to support participatory monitoring of biodiversity [41]. Fordham et al. [11] summarized two community monitoring projects in Brazil that can potentially be incorporated into MRV in REDD+ context, but publications that provide further detail on these projects were not found. Nearly half of the early REDD+ projects in Brazil have been implemented as local Payment for Ecosystem Service (PES) schemes [40] so participatory monitoring activities may be conducted in PES context. Meanwhile, a review of sub-national REDD+ projects in Mexico found that PMRV implementation can be limited by the capacity to conduct MRV despite the participation of local actors in initiating local REDD+ projects [27]. Integrating PMRV activities into existing participatory monitoring activities may improve local capacity to meet MRV requirements and maintaining the high level engagement of local communities.

The rapid increase in the number of PMRV publication over time, which culminated with a special issue of the journal *Forests* in 2014/2015 that published 8 PMRV studies, has not been matched with the rate of PMRV implementation in forest carbon projects. Danielsen et al. [9] estimated that 52% of forest carbon projects validated by Climate, Community and Biodiversity Alliance (CCBA) involved local stakeholders in 2012, with increased participation of local stakeholders in monitoring biomass, biodiversity and livelihood over time. PMRV projects has mostly been aimed to assess the feasibility of PMRV, which often limited the participation of local communities to data gathering. Shrestha et al. [31] have shown that embedding PMRV into a broader local implementation of REDD+ created more opportunities to assess REDD+ social outcomes, such as equity in benefit sharing, safeguard implementation and improved forest governance, as well as increasing participation level of local communities.

Three empirical PMRV studies have been published as NPRP publications [12, 15, 25]. Although the results of these studies have not been analyzed with quantitative or statistical methods, they still contribute valuable field-based experiences of PMRV implementation. For examples, these studies have shown that local communities can obtain a range of monitoring data that include biomass, resource use and biodiversity [15, 25]; while well-defined land tenure and access to resources encourages participation [12, 25]. This indicates that the type of publication, PRP or NPRP, does not reflect the division between empirical and non empirical studies in PMRV research. However, it suggests that there may still be barriers (e.g. capacity, cost, priority) for those implementing PMRV, particularly non-government organizations, to publish their results as peer-reviewed publications.

As the majority of studies on PMRV focus on measuring carbon stocks, there are very few examples or analyses of the involvement of local communities in reporting. Consistent reporting process at the local level can be difficult to achieve [25]. Boissière et al. [21] suggests that existing reporting structures in other sectors may provide examples for a participatory reporting framework, such as community participation in information flows within the health sector in Indonesia. Other publications have advocated for the incorporation of local data into a nested MRV system [6, 20, 34, 36], in which data are collected and reported across multiple levels of governance from local to national. Although a few models of participatory reporting framework have been proposed [20, 34], they are yet to be tested.

Several PMRV publications have found that the use of technology, such as a smart phones and personal digital assistants (PDA) linked to a global positioning system (GPS) with GIS, can simplify the documentation and reporting of PMRV data [5, 6, 11, 14, 18, 22, 25, 36, 37, 42]. However, there is also concern that dependence on networked electronic devices carries the risks of frequent equipment failures in remote settings and of improper use [5, 21]. A recent review found digital data entry can contribute to the success of participatory monitoring when combined with stakeholder participation, but it may have detrimental effects on the sustainability of the monitoring if it is not effectively implemented [43]. Thus, the increased ease of reporting gained from the use of those devices should be considered against the risks.

The verification process in MRV is assigned to an external, independent third party [44], without any input from local communities [9, 34]. In addition to this, the procedures to verify carbon emissions are complex [44]. Some studies suggest that local communities can participate in verification through a range of activities to verify remote sensing data, such as documenting forest change events [11, 25, 26] and participatory mapping of forest strata and land use [15, 21]. However, these activities have also been considered as participatory measurement activities that can contribute to internal verification of MRV data at project and national levels [5, 9, 14, 15, 24–26, 34]. Thus, participation of local communities in verification may be limited to collecting verification data rather than conducting independent verification.

### Empirical evidence supporting PMRV claims

The number of EFSE supporting a PMRV claim reflects the significance, feasibility and measurability of REDD+ outcomes associated with the claim. The two PMRV claims with the highest number of EFSE were lowering the cost of MRV and accuracy of local measurements (Fig 4), which indicate the importance of those outcomes in supporting the MRV system and REDD+ implementation and the feasibility of achieving them. Meanwhile, PMRV claims of REDD+ social outcomes have been supported with fewer number of EFSE than PMRV claims of REDD+ technical outcomes (Fig 4). This may be due to the difficulties in measuring those outcomes, lead time for those outcomes to be realized and limited opportunities to test them. Several components of REDD+, such as benefit distribution models [19] and safeguard indicators [7, 45, 46], are still being debated and are yet to be fully developed and implemented. In addition, REDD+ social outcomes were also supported with less CSE and AS that suggest that they received less attention than REDD+ technical outcomes. Given that many REDD+ social outcomes directly affect local communities, measures and indicators to assess those outcomes need to be developed further and incorporated into future PMRV studies.

In this study, we defined EFSE to include quantitative and qualitative evidence presented in the analyzed publications. For example, EFSE supporting cost effectiveness of PMRV was based on quantitative cost analysis [8–10, 13, 14], while EFSE supporting increased engagement or ownership was based on qualitative observation [24, 26]. We acknowledge that variations in sample size and method used in PMRV studies may affect the robustness of quantitative evidence (Table 2), while there may be some subjectivity in categorizing qualitative evidence. Several PMRV projects [24, 31] collected data that can be used as indicators to assess REDD+ social outcomes, such as enhancement of co-benefits or improved forest management. This suggests that some REDD+ social outcomes can be supported with quantitative evidence. Meanwhile, qualitative evidence and analysis may be required to assess other social outcomes.

Adopting lessons from non-PMRV contexts may be helpful in the early stages of PMRV development when the implementation of PMRV is still limited. However, there is a need to clarify whether the lessons from those contexts are directly applicable to PMRV. For example, the claim of the potential for inaccurate reporting of carbon stocks due to the link between financial rewards and performance [9, 19, 37] has been based on citations to several studies conducted in the context of participatory monitoring of biodiversity and natural resources [47, 48]. In the PMRV context, this risk is minimized because independent verification is an integral part of MRV [9, 19, 22] and there are simple methods to verify local measurements [22]. Data collection and reporting in the MRV context must follow specific guidelines [3], while there is a scope for some discretion in determining monitoring subjects and procedures in other participatory initiatives. Lessons from non-PMRV contexts can be relevant when the enabling conditions can be replicated in PMRV, such as local communities can contribute local knowledge that may improve data quality [6, 9, 10, 13, 15, 23–26, 33, 34, 36–38]. Thus, applicability of lessons from other participatory contexts may depend on the ability to replicate the enabling conditions.

### Contradictory PMRV claims

We have identified PMRV claims that potentially hinder or present challenges to achieve the desired REDD+ outcomes. These contradicting claims do not negate the positive PMRV claims, but they indicate the risks or conditions that may compromise the desired REDD+ outcomes. For example, cost effectiveness of PMRV in obtaining local data has often been used to justify PMRV implementation [8–10, 13, 14, 22], but opportunity cost can exceed the benefit for the local communities [13, 14, 24, 25, 31]. Similarly, there were challenges in achieving REDD+ outcomes such as measurement accuracy, increased local engagement and equity in benefit sharing (see Table 3). Addressing the factors that contribute to the risks may help eliminate them.

### Study limitations

Our literature search results are limited by the databases that we have used and selected language (English), which may not include all published PMRV literature. Several websites with publication collections, such as CIFOR and Community Carbon Forestry, have institutional links with particular research teams. This can result in a disproportionately high number of their publications in the search results. As most of the relevant PMRV publications from those collections are also listed in other databases, we found that those collections have not introduced a bias towards certain researchers in the search results. Meanwhile, the use of references or citation of articles to identify additional publications may limit the analysis by selecting publications that promote similar ideas or views. We employed this method to identify seven out of 29 PMRV publications.

We found that multiple publications may report the same outcomes from the same research project. This presents a problem as including these publications in the analysis would result in inflating the number of certain PMRV claims. The challenge was in identifying and excluding the duplicates from the analysis of PMRV publications. For example, Global Canopy Programme published a policy brief and a report on the progress of community forest monitoring projects. We included the policy brief [11] in our analysis, but excluded the report. Similarly, we identified multiple publications that report the outcomes of the K:TGAL project in the literature search so we selected publications that report the results from different countries involved in that project [16, 22].

Identifying PMRV claims is easier in an empirical study than in a non-empirical one as the claims (i.e. the effects of PMRV on REDD+ outcomes) can usually be found in the introduction, result or discussion sections. Non- empirical study does not have a fixed structure so there is a greater risk of missing a PMRV claim in the text. To minimize the risk, we analyzed those publications more than once. Multiple occurrences of the same claim in the text are examined carefully to ensure the claim is registered only once with the appropriate supporting evidence (CSE and/or EFSE or AS). In analyzing the supporting evidence for PMRV claims, the number of PMRV claims with assumption may have been over-estimated. A failure to provide explicit citation to a PMRV claim would result in the claim being assigned with assumption instead of citation. This may occur in non peer-reviewed publications as there may be less emphasis on citations in some publication types (e.g. policy briefs). Qualitative evidence was also more difficult to identify than quantitative evidence.

## Conclusions

We have identified 29 PMRV publications in this literature review, consisting 20 peer-reviewed and 9 non peer-reviewed studies. Empirical studies made up nearly half of all publications. PMRV projects reported in the empirical studies were conducted in 17 countries across three tropical continents, forest and land tenure types. However, only two PMRV projects were located in Latin America, where early REDD+ projects have been implemented mainly as PES projects. Most PMRV projects tested the feasibility of PMRV, particularly participatory measurement or monitoring, which often limited the participation of local communities to data gathering. Development and implementation of participatory reporting frameworks have been lacking despite the need for integrating local data to the national database.

Recent PMRV projects yielded empirical evidence that PMRV can support and enhance REDD+ outcomes, although types and robustness of evidence varied amongst studies. In particular, PMRV can yield accurate local biomass data when adequate training and support are provided, while lowering the cost of MRV when benefit for local communities exceeds opportunity cost. PMRV claims of achieving REDD+ social outcomes, such as increased environmental awareness and equity in benefit sharing, were generally supported by less empirical evidence than REDD+ technical outcomes. This may be due to the difficulties in measuring those outcomes and the slow progress in the development and implementation of REDD+ components. Lessons from other monitoring contexts can be useful to predict the effects of PMRV or develop its implementation, but only when the enabling conditions can be replicated in REDD+ contexts.

Robust empirical evidence on the effects of PMRV on REDD+ outcomes provides valuable information on whether PMRV should be implemented and improve Its ability to maximize positive REDD+ outcomes. The following factors should be considered in future PMRV studies in order to address current knowledge gaps. Integrating PMRV study into a local REDD+ implementation may provide more opportunities to assess REDD+ outcomes that affect local communities directly, such as safeguard implementation, benefit sharing, quality of governance and enhanced co-benefits. This can also increase participation level of local communities beyond data gathering through their engagement as local stakeholders of REDD+. More rigorous assessments of REDD+ social outcomes are needed. Participatory reporting framework should be further developed and tested to ensure that local MRV data can be incorporated into national databases. Empirical studies, along with their analysis and data, are important sources of empirical evidence of PMRV effects on REDD+ and local communities so their publication should be encouraged and facilitated.

## Supporting Information

S1 Table Preferred Reporting Items for Systematic Reviews and Meta Analyses (PRISMA) checklist. (DOCX)
